# Evaluation of a steroid delivery system to mitigate the severity of proliferative vitreoretinopathy in a minipig model

**DOI:** 10.3389/fopht.2023.1222689

**Published:** 2023-11-17

**Authors:** Chee Wai Wong, Ning Cheung, James S. Howden, Joanna Marie Fianza Busoy, Shaun Sim, Joshua Lim, Candice Ho, Amutha Barathi Veluchamy, Gert Storm, Tina T. Wong

**Affiliations:** ^1^ Singapore National Eye Centre (SNEC), Singapore, Singapore; ^2^ Singapore Eye Research Institute, Singapore, Singapore; ^3^ Department of Pharmaceutics, Utrecht Institute for Pharmaceutical Sciences (UIPS), Utrecht University, Utrecht, Netherlands; ^4^ Department of Experimental Molecular Imaging, University Clinic and Helmholtz Institute for Biomedical Engineering, RWTH Aachen University, Aachen, Germany

**Keywords:** proliferative vitreoretinopathy, liposome, drug delivery, retinal detachment, animal model

## Abstract

**Purpose:**

To investigate the efficacy of liposomal prednisolone phosphate to mitigate the severity of proliferative vitreoretinopathy (PVR) in a minipig model of PVR.

**Methods:**

A total of 18 eyes of 9 minipigs underwent PVR induction surgically. Eyes were randomized equally into three groups: intravitreal injection of liposomal prednisolone phosphate (LPP), triamcinolone acetonide (TA), and controls. PVR severity was graded on fundoscopic examination using a modified version of the Silicon Study Classification System. Severe PVR was defined as grade 2-5 on this classification, and the proportion of eyes with retinal detachment from severe PVR, defined as retinal re-detachment, i.e., PVR grade 2-5, was compared between treatment and control groups.

**Results:**

On day 28, five eyes (83.3%) in the control group were observed to have severe PVR. Within the LPP group, one (16.7%) eye developed retinal detachment due to severe PVR. Grade 0 PVR was observed in four (66.7%) eyes, grade 1 in one (16.7%) eye, and grade 5 in one (16.7%) eye. Within the TA group, grade 0 PVR was observed in four eyes (66.7%), grade 1 in two eyes (16.6%), and grade 5 in one (16.7%) eye. The difference in the proportion of eyes with severe PVR was significantly lower in the LPP group compared to controls at day 28 (16.7% vs 83.3%, p=0.02). There was no significant difference in the rate of severe PVR or median PVR grade between the liposomal prednisolone phosphate and triamcinolone acetonide groups.

**Conclusion:**

Liposomal prednisolone phosphate reduces the severity of PVR in a minipig model of PVR.

## Introduction

Proliferative vitreoretinopathy (PVR) is a blinding condition that can occur secondary to penetrating ocular trauma or rhegmatogenous retinal detachment (RRD). This process can result in massive tractional detachment of the retina and intraretinal scarring leading to loss of vision (1). The anatomical success rate of surgery for RRD complicated by PVR is only 69-75% compared to 98% in RRD without PVR (2, 3). When PVR is present, multiple surgeries are often required to reattach the retina, frequently with unsatisfactory visual outcomes (3). In addition, patients with PVR incur twice the amount of healthcare resources compared to patients without PVR (3).

Adjunctive pharmacological treatments for PVR have shown promising results in animal models of PVR but have so far yielded less satisfactory outcomes in clinical trials (4–12). Inflammation is a well-established step in PVR pathogenesis (13, 14). Thus, steroids, with their anti-inflammatory properties, would appear to be an ideal solution for the treatment of PVR, yet clinical results have not been satisfactory. The exact reasons for the lack of efficacy are not well understood but could be related to a lack of specificity for the inflammatory cell types in PVR, insufficient drug concentration to reach therapeutic thresholds, inability to maintain therapeutic levels for a sustained duration, suboptimal timing of treatment, and patient selection. Some of these problems can be circumvented by the use of a targeted drug delivery system. Liposomal encapsulation of glucocorticoids has several beneficial effects, including the enhancement of the therapeutic index by prolonging half-life, and avoidance of toxic effects from unintended action at non-inflamed sites (15). The goal of this study is, therefore, to investigate the efficacy of a small-sized liposomal prednisolone phosphate preparation as a treatment to mitigate the severity of PVR developed in an experimental minipig model of PVR.

## Methods

The SingHealth Institute Animal Care and Use Committee (IACUC, Singhealth Approval Number 2019/SHS/1502) gave approval for this study. All procedures were conducted in accordance with the ARVO Statement for the Use of Animals in Ophthalmic and Vision Research. A total of 18 eyes of 9 male Gottingen minipigs were used for this study. The age range was 1 year ± 3 months and the weight range was 15-25kg. All minipigs were healthy with no evidence of any comorbidities. The minipigs were anesthetized with an intraperitoneal injection of ketamine hydrochloride (35- 50mg/kg) and xylazine (5- 10mg/kg) prior to induction of PVR. Topical anaesthesia (proparacaine hydrochloride 0.5%) was applied and pupils were dilated with tropicamide 1%.

### Liposomal prednisolone phosphate

LPP is a liposomal formulation of prednisolone phosphate containing dipalmitoyl phosphatidyl choline (DPPC), cholesterol, and PEG2000 distearoyl phosphatidylethanolamine (PEG-DSPE) in a 62%, 33%, and 5% molar ratio, respectively. Liposomes were sized at 110nm with a polydispersity index (PDI) of 0.04, zeta potential of +4.3 meV, and concentration of 5mg/ml of prednisolone phosphate (16).

### Platelet-rich plasma preparation

Platelet-rich plasma contains cytokines, including VEGF and PDGF, that support the inflammation, proliferation, and remodeling that are essential in the pathogenesis of PVR (17). In both procedures, platelet-rich plasma (PRP) was prepared from minipig homologous blood 30 minutes prior to surgery, according to the method explained by Constable et al. (18) Arterial blood was collected from the minipig’s ear artery into citrate blood tubes (one part 3.8% sodium citrate to nine parts whole blood). The blood was centrifuged at 1,200 rotations per minute for 10 minutes, and the upper third of the supernatant PRP was aspirated. Platelet counts were performed on an automated Coulter counter to achieve 600,000 platelets per cubic millimeter. Then, 0.1ml of PRP was injected into the vitreous cavity at the end of surgery.

### Induction of PVR

PVR was induced based on the method previously described by our group (19). Briefly, a three-port valved 25-gauge pars plana vitrectomy (Constellation; Alcon) was performed, 3mm from the limbus, taking care to avoid traumatizing the lens. First, posterior vitreous detachment was induced by suction, followed by core vitrectomy and shaving of the peripheral vitreous. Then, RPE detachment was induced in the inferior retina by injecting a balanced salt solution (Alcon Laboratories, Fort Worth, TX) into the sub-RPE space, using a 39-gauge angled cannula. A retinotomy of 3-disc diameters was created within this area of RPE detachment with the vitrector. A 39-gauge angled cannula was then advanced into the sub-RPE space to scrape the RPE and dislodge these cells into the vitreous cavity. A 90% fluid air exchange was performed to disrupt any remaining vitreous and to allow RPE cells to settle onto the retinal surface. Finally, 0.1 mL of PRP was injected into the vitreous cavity, and eyes randomized to treatment groups received either 0.1ml of 5mg/ml liposomal prednisolone phosphate or 0.1ml of 4mg/ml triamcinolone acetonide. All procedures were performed by a single surgeon (CWW). Topical Tobramycin was administered four times a day for 5 days after induction of PVR.

### Investigations and examination

Dilated slit lamp examination was performed by two double-masked ophthalmologists to assess the lens status. Intraocular pressure was measured with contact tonometry (Tono-pen, Reichet, NY, USA). The retina was examined with an indirect ophthalmoscope through a +20 D fundus lens on days 1, 7, 14, 21, and 28 by two double-masked ophthalmologists. We used a modified version of the Silicon Study Classification System, a classification of human PVR, that was also employed by Umazume’s group (20) in their description of a swine model of PVR:

Grade 0: Normal retina, retinal or vitreous pigment clumps
Grade 1: Inner retinal wrinkling
Grade 2: Retinal detachment, 1 quadrant (1-3 clock hours)
Grade 3: Retinal detachment, 2 quadrants (4-6 clock hours)
Grade 4: Retinal detachment, 3 quadrants (7-9 clock hours)
Grade 5: Retinal detachment, 4 quadrants (10-12 clock hours)

Severe PVR was defined as retinal re-detachment, i.e., grade 2-5. Fundus photographs and optical coherence tomography (OCT) were performed using a spectral domain (SD)-OCT system (Spectralis, Heidelberg Engineering Inc. Germany). At the end of the study, minipigs were euthanized with intraperitoneal pentobarbitone (60 - 150mg/kg), and study eyes were enucleated.

### Collection of vitreous samples and analysis

Vitreous humor samples of at least 0.2ml were obtained at the start of the vitrectomy procedure with the vitrector attached to a 3 ml syringe. On day 28, the eyes were enucleated and the vitreous was obtained prior to paraffin fixation of the eye. Vitreous samples were stored at -80 degrees Celsius prior to analysis. The vitreous concentrations of interleukin 6 (IL-6), C reactive protein; (CRP), platelet-derived growth factor BB (PDGF-BB), placental growth factor (PLGF), and vascular endothelial growth factor isoform A (VEGF-A) were determined using the Human multiplex ELISA kit from AYOXXA (Ayoxxa biosystems, Cologne, Germany).

### Histopathology and immunohistochemistry

Whole minipig eyes (28 days, n=12) were enucleated and fixed in a mixture of 10% neutral buffered formalin solution (Leica Surgipath, Leica Biosystems Richmond, Inc.) for 48 hours. The whole eyes were then dissected to anterior and posterior segments prior to dehydration in increasing concentration of ethanol, clearance in xylene, and embedding in paraffin (Leica-Surgipath, Leica Biosystems Richmond, Inc.) Four-micron sections were cut with a rotary microtome (RM2255, Leica Biosystems Nussloch GmbH, Germany) and collected on POLYSINE™ microscope glass slides (Gerhard Menzel, Thermo Fisher Scientific, Newington, CT). The sections were dried in an oven at a temperature of 37°C for at least 24 hours. To prepare the sections for histopathological and immunofluorescence examination, the sections were heated on a 60°C heat plate, deparaffinized in xylene, and rehydrated in decreasing concentration of ethanol. A standard procedure for Hematoxylin and Eosin (H&E) was performed. A light microscope (Axioplan 2; Carl Zeiss Meditec GmbH, Oberkochen, Germany) was used to examine the slides, and images were captured.

In parallel, immunofluorescence staining was performed. Heat-induced antigen retrieval was performed by incubating sections in sodium citrate buffer (10 mM Sodium citrate, 0.05% Tween 20, pH 6.0) for 20 minutes at 95-100°C. The sections were then cooled down in sodium citrate buffer for 20 minutes in RT and washed three times for 5 minutes each with 1X PBS. Non-specific sites were blocked with a blocking solution of 5% bovine serum albumin (BSA) in 0.1% Triton X-100 and 1XPBS for 1 hour at room temperature in a humidified chamber. The slides were then rinsed briefly with 1X PBS. A specific primary antibody, shown in **Table 1**, was applied and incubated overnight at 4°C in a humidified chamber prepared in blocking solution. After washing twice with 1XPBS and once with 1X PBS with 0.1% tween for 10 minutes each, Alexa Fluro® 488 – conjugated fluorescein-labeled secondary antibody, shown in **Table 1** (Invitrogen- Molecular Probes, Eugene, OR), was applied at a concentration of 1:1000 in blocking solution and incubated for 90 minutes at RT. The slides were then washed twice with 1XPBS and once with 1X PBS with 0.1% tween for 5 minutes each, the slides were mounted on the slides with Prolong Diamond Anti-fade DAPI5 Mounting Media (Invitrogen- Molecular Probes, Eugene, OR) to visualize cell nuclei. For negative controls, the primary antibody was omitted.

**Table 1:** Primary antibodies used for immunohistochemical staining.

Antibody	Catalog No.	Company	Concentration
Alpha smooth muscle actin	710487	Thermo Fisher Scientific	1:200
Glial Fibrillary Acid Protein GA5	14-9892-82	Thermo Fisher Scientific	1:200
Macrophage, RAM11	M0633	DAKO	1:20
Alexa Fluor 488 goat anti−mouse IgG (H+L)	A11001	Invitrogen. Life Technologies (Invitrogen, Eugene, OR)	1:1000
Alexa Fluor 488 goat anti−rabbit IgG (H+L)	A11008	Invitrogen. Life Technologies (Invitrogen, Eugene, OR)	1:1000
Alexa Fluor 488 donkey anti−goat IgG (H+L)	A11055	Invitrogen. Life Technologies (Invitrogen, Eugene, OR)	1:1000

A fluorescence microscope (Axioplan 2; Carl Zeiss Meditec GmbH, Oberkochen, Germany) was used to examine the slides, and images were captured. Experiments were repeated in duplicates for the antibody.

### Statistics

Six minipigs in each group were required to achieve a power of 80% and a level of significance of 5% (two-sided) for detecting a clinically significant effect size of 2 between treatment and control eyes. The Chi-square analysis (two-sided) was used to compare the proportion of eyes with severe PVR between groups. Kruskal Wallis test was used to compare median PVR grades between groups. Statistical significance was set at p=0.05 and Stata version 16.0 was used for the analysis.

## Results

### PVR grading

**Figure 1:**
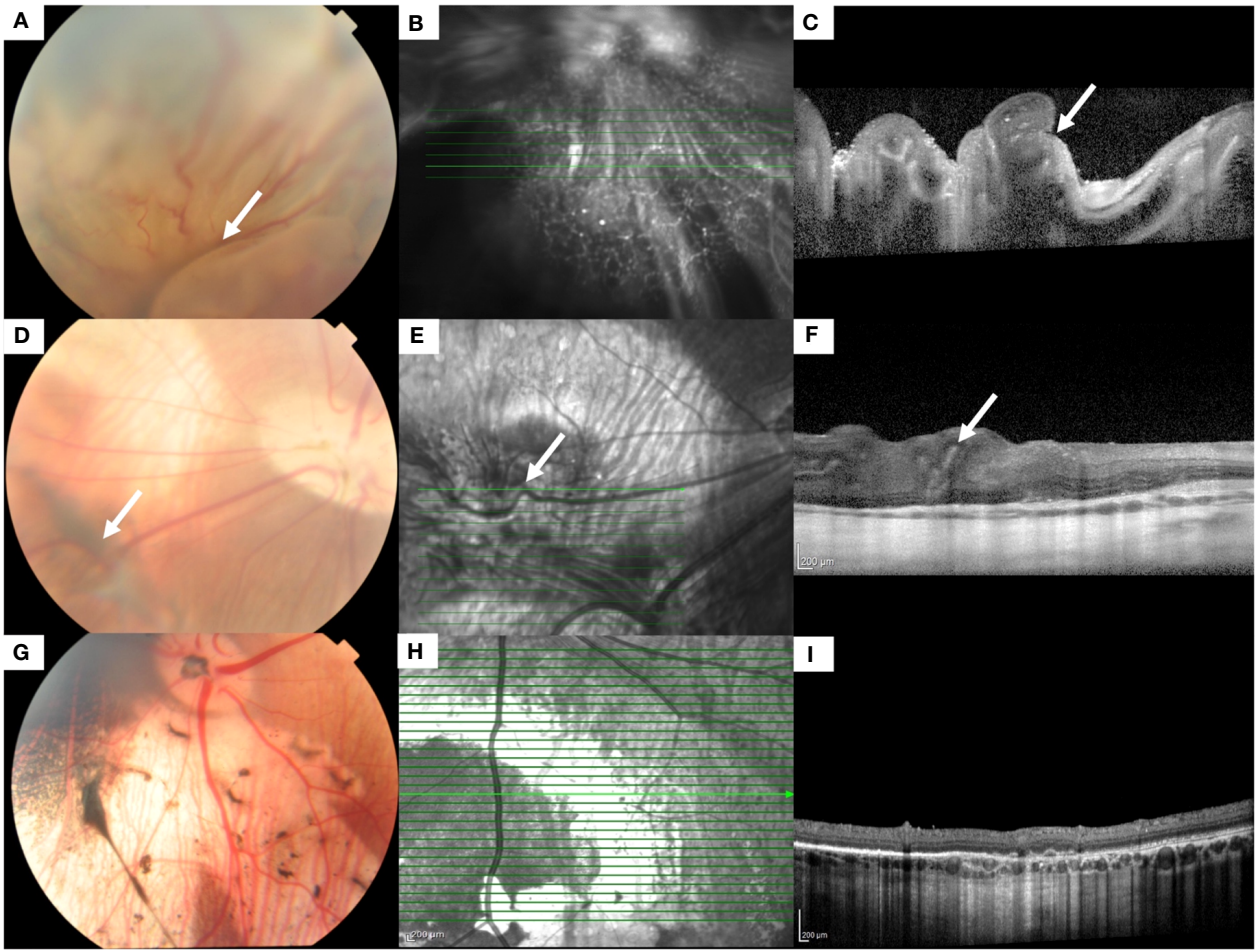
Fundus color photographs and optical coherence tomographic (OCT) scans of an eye with grade 5 PVR **(A-C)** and grade 1 PVR **(D-F)**. **(A)** Fundus photograph shows a totally detached retina with folds (white arrow). **(B)** Infrared en face image of the detached retina. The highlighted green line indicates the scan segment shown in **(C)**, where the folding of the detached retina (white arrow) can be seen. **(D)** Fundus photograph shows the attached retina with epiretinal/intraretinal pucker (white arrow). **(E)** Infrared en face image shows the distortion of a retinal vessel (white arrow) crossing the area of the pucker. The highlighted green line indicates the scan segment shown in **(F)**, where distortion of the inner retina (white arrow) can be seen. **(G-I)** Fundus color photograph, infrared en face and OCT image of an eye with no PVR.

On day 7, the fundal view was obscured in nine eyes (two in the control group, three in the liposomal prednisolone phosphate group, and four in the triamcinolone acetonide group) due to vitreous hemorrhage. By day 14, vitreous hemorrhage had resolved, and fundal examination was possible in all eyes. On day 28, five eyes (83.3%) in the control group were observed to have severe PVR and detachment of the retina. Within the control group, PVR grade 1 (**Figure 1**) was observed in one (16.7%) eye, grade 2 in one (16.7%) eye, grade 3 in two (33.3%) eyes, and grade 5 (**Figure 1**) in two (33.3%) eyes. Within the liposomal prednisolone phosphate group, only one (16.7%) eye developed retinal detachment due to severe PVR. Grade 0 PVR was observed in four (66.7%) eyes, grade 1 in one (16.7%) eye, and grade 5 in one (16.7%) eye. Within the triamcinolone acetonide group, grade 0 PVR was observed in four eyes (66.7%), grade 1 in two eyes (16.6%), and grade 5 in one (16.7%) eye. The difference in the proportion of eyes with severe PVR was significantly lower in the liposomal prednisolone phosphate group compared to the control group at day 28 (16.7% vs 83.3%, p=0.02). The median PVR grade was also significantly lower in the liposomal prednisolone phosphate group than the control group at day 28 (0 vs 3, p=0.039). There was no significant difference in the rate of severe PVR or median PVR grade between the liposomal prednisolone phosphate and triamcinolone acetonide groups. **Table 2** compares the PVR staging at all time points for treatment and control groups.

**Table 2:** Median PVR grade in treatment and control groups.

Day	Median PVR grade			P*
	Liposomal prednisolone phosphate (n=6)	Triamcinolone acetonide (n=6)	Control group (n=6)	
0	0	0	0	1.0
7	0	0	0	0.39
14	0	0	1	0.11
21	0	0.5	1	0.15
28	0	0.5	3	0.039

*Kruskal Wallis test comparing median PVR grade between treatment and control groups.

### Cytokine and growth factor levels

VEGF-A levels were higher in the control group on day 28 compared to the liposomal prednisolone phosphate group (895.6 ± 1239.4 vs 433.0 ± 204.5, p=0.29), but this difference did not reach statistical significance. Mean levels of IL-6, CRP, PDGF-BB, and PLGF were similar between the three groups at day 28, and between baseline and day 28 within each group (**Table 3**).

**Table 3:** A comparison of cytokine and growth factor levels between treatment and control groups.

Cytokine (pg/ml)	Liposomal prednisolone phosphate			Triamcinolone acetonide			Control			P †	
	Day 0	Day 28	P*	Day 0	Day 28	P*	Day 0	Day 28	P*	Day 0	Day 28
IL-6	3.6 ± 0.8	3.9± 1.1	0.90	2.0 ± 0.3	1.4 ± 0.53	0.16	3.4 ± 1.8	3.4 ± 0.9	0.59	0.82	0.54
CRP	68.4± 24.3	72.5 ± 22.6	0.91	50.8 ± 14.3	68.3 ± 48.7	0.99	58.4 ± 36.1	50.1 ± 20.4	0.56	0.64	0.14
PDGF-BB	11.7 ± 6.5	19.9 ± 14.3	0.12	15.6 ± 10.3	23.0 ± 17.6	0.49	12.5 ± 7.6	20.0 ± 16.3	0.25	0.81	0.99
VEGF-A	318.0 ± 95.5	174.8 ± 68.9	0.10	421.9 ± 169.6	447.9 ± 313.8	0.89	433.0 ± 204.5	895.6 ± 1239.4	0.40	0.24	0.29
PLGF	14.2 ± 3.6	22.8 ± 30.3	0.50	22.8 ± 8.3	26.2 ± 16.7	0.71	18.5 ± 4.6	18.9 ± 20.1	0.96	0.11	0.80

*paired t-test comparing mean levels of each cytokine at baseline and at day 28.

†One-way ANOVA comparing mean levels of cytokines between treatment and control groups.

IL-6, interleukin 6; CRP, C reactive protein; PDGF-BB, platelet-derived growth factor-BB isoform; VEGF-A, vascular endothelial growth factor isoform A; PLGF, placental growth factor.

### Adverse events

Cataracts and elevated intraocular pressure (IOP) are well-known complications of intravitreal steroid treatment. At the end of the study, none of the eyes in the liposomal prednisolone phosphate group or triamcinolone acetonide groups had developed cataracts. Mean intraocular pressure was 16± 3.8mmHg, 17± 4.6mmHg, and 15± 4.2mmHg (p=0.89) in the liposomal prednisolone phosphate, triamcinolone acetonide, and control groups, respectively. No spike in IOP above 21mmHg was observed throughout the duration of the study in both the TA and LPP groups.

### Histology and immunohistochemistry

**Figure 2:**
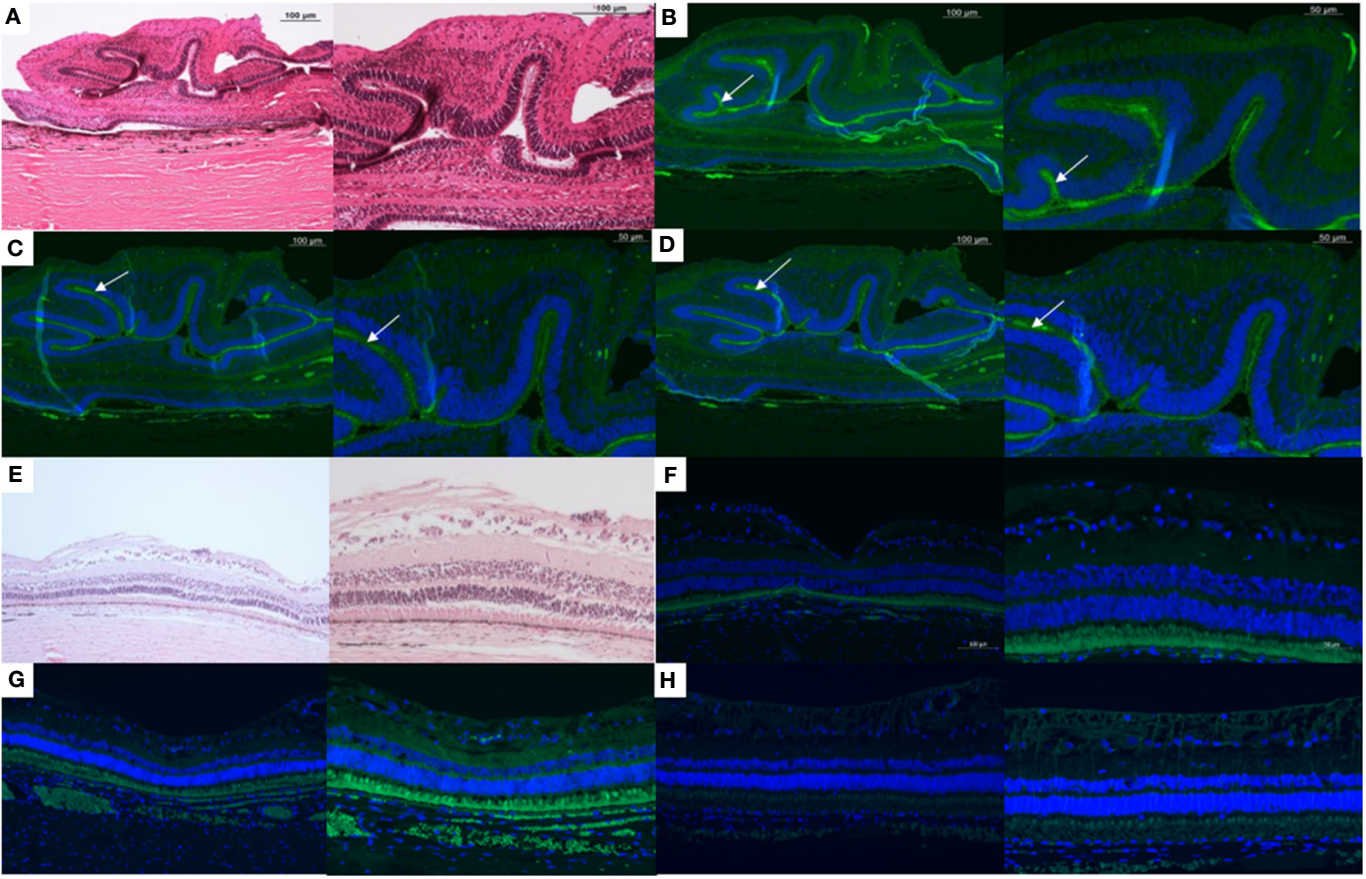
Histology and immunohistochemistry slides of a minipig eye with severe proliferative vitreoretinopathy (PVR) shown at 10x (left) and 20x (right) magnification. **(A)** H&E staining demonstrates folding of the retina, a characteristic of PVR. Positive staining for the following (white arrows) can be seen associated with the subretinal surface of these retinal folds: **(B)** Macrophages (RAM11 stain), a major inflammatory cell type involved in the PVR process, **(C)** Alpha smooth muscle actin, a marker for myofibroblasts derived mainly from de-differentiated RPE cells, and **(D)** Glial fibrillary acid protein (GFAP), a marker of glial cells. E-H:10x (left) and 20x (right) magnification of the H&E **(E)**, RAM11 **(F)**, alpha smooth muscle actin **(G)**, and GFAP **(H)** staining of a minipig eye without PVR.

H&E staining demonstrated folding of the retina, a characteristic of PVR (**Figure 2A**). Positive staining for the following could be seen associated with the retinal folds: (**Figure 2B**) Macrophages, a major inflammatory cell type involved in the PVR process, (**Figure 2C**) Alpha smooth muscle actin, a marker for myofibroblasts derived mainly from de-differentiated RPE cells, and (**Figure 2D**) Glial fibrillary acid protein (GFAP), a marker of glial cells. **Figures 2E–H** show the histology and immunohistochemical staining of an eye without PVR.

## Discussion

This is the first study evaluating the effectiveness of liposomes as a drug delivery system for steroids to treat PVR. Eyes that received liposomal prednisolone phosphate were less likely to develop retinal re-detachment from severe PVR and had significantly lower median PVR staging at 4 weeks after PVR induction. Intravitreal cytokine and growth factor levels were similar in all groups at day 28, except for VEGF-A, which was higher in the control group but did not reach statistical significance. In the following sections, we discuss the role of inflammation in PVR, the evidence for steroids as an adjunctive treatment of PVR, why it has not worked in the past, and provide a hypothesis for the efficacy of liposomal prednisolone phosphate observed in our study.

Following retinal detachment, a cascade of cellular events occurs to facilitate retinal repair. Inflammation is believed to be a key driver of this process. In particular, clinical risk factors of PVR suggest that when inflammation exceeds an as-yet-unknown threshold in eyes with retinal detachment, the balance is tipped from retinal repair to PVR (13). Briefly, these sequences of events begin with a breakdown in the blood-retinal layer, exposing the immune-privileged intraocular environment to macrophages. Macrophages not only release pro-inflammatory elements but also promote photoreceptor cell death by apoptosis through monocyte chemoattractant protein-1 (MCP-1). At the same time, sustained separation of the neurosensory retina leads to ischemia and neuronal cell death, and glial elements including microglia and Müller cells promote retinal remodeling to replace dying photoreceptors, which eventually leads to retinal shortening (intraretinal PVR). Müller cells extend their processes into the subretinal space where they interact with macrophages, microglia, and RPE cells to form subretinal membranes (subretinal PVR). RPE cells detach from the Bruch’s membrane and migrate through retinal breaks into the vitreous cavity, where they undergo an epithelial-mesenchymal transition (EMT). In this process, EMT-inducing transcription factors induce the expression of genes that maintain the mesenchymal state and suppress the epithelial state. Cell surface epithelial cadherin (E-cadherin) molecules on RPE cells, which normally maintain the structural integrity of the RPE monolayer, are suppressed, and RPE cells gain a spindle-shaped morphology and express markers associated with mesenchymal cells, such as neural cadherin (N-cadherin) (13). A detailed discussion of the various pathways of EMT induction is beyond the scope of this discussion, but relevant to inflammation is the role of macrophages. In lung carcinoma cells, macrophages secrete Il-6, which is responsible for inducing EMT by activating the cyclooxygenase 2 (COX2), prostaglandin E2 (PGE2), and beta-catenin signaling pathways. Subsequently, EMT-induced immunosuppressive effects have been observed which include, in part, the recruitment of M2 macrophages that secrete immunosuppressive, angiogenic, and chemotactic factors, which further enhance the survival, proliferation, and migration of the transformed mesenchymal cells (21). Whether M2 macrophage recruitment occurs in PVR requires further study but may explain why anti-inflammatory treatment after EMT has already occurred (i.e., in PVR grade C or worse) is less ineffective, as discussed in the following section.

The potential of steroid treatment for PVR has been well investigated, with a number of studies conducted evaluating triamcinolone acetonide (TA) or dexamethasone, given intravitreally, for improving anatomical outcomes in patients with PVR with generally small effect sizes (5, 6, 8–12). **Table 4** summarizes these studies. In a randomized controlled trial (n=75), Ahmadieh et al. injected intravitreal TA 4mg in eyes with PVR grade C or worse and found a single surgery success rate of 84.2% compared to 78.4% in controls at 6 months (p=0.5) (9). Acar et al. conducted a case-control study (n=72) in which eyes with PVR grade C or worse received adjunctive intravitreal TA 4mg during surgery (group 1) or did not receive any steroid (group 2). Re-detachment rates were marginally lower (12.5%) in group 1 compared to group 2 (21.87%, P = 0.349) (8). These studies show that intravitreal TA in eyes with PVR grade C appeared to have a statistically insignificant benefit in terms of anatomical outcomes. A more potent steroid may produce a greater clinical effect. Dexamethasone has five times greater potency than TA and may achieve higher vitreous concentrations due to its higher hydrophilicity but suffers from a short half-life, thus necessitating a sustained release drug delivery system (5). In a more recent study, the efficacy of a slow-release dexamethasone (700ug) implant (Ozurdex) injected intravitreally in eyes with PVR grade C was investigated in a randomised controlled trial enrolling 140 patients. Again, anatomic success at 6 months did not differ between the intervention and control groups (49.3% vs. 46.3%, p=0.73). However, Ozurdex significantly reduced the incidence of cystoid macular edema in treated eyes vs controls (42.7% vs 67.2%, p=0.004), thus demonstrating some anatomical benefit in these eyes (5). A possible reason for the small effect size in these studies could be that inflammation is the predominant event in early PVR but plays a relatively minor role in later stages where cellular proliferation and EMT become the major processes. A trial conducted by Asaria et al. in 2001 provided some basis for this hypothesis (22). This was a randomised placebo-controlled clinical trial involving 174 participants with rhegmatogenous retinal detachment viewed to be at high risk of developing postoperative PVR (i.e., pre-PVR patients). The treatment group was given a continuous intraocular low molecular weight heparin (LMWH) and 5-fluorouracil (5-FU) infusion during surgery to repair the retinal detachment. The incidence of postoperative PVR was significantly lower in the treatment group than in the placebo group (12.6% vs 26.4%, p=0.02). LMWH exerts anti-inflammatory action via inhibition of pro-inflammatory cytokines while 5-FU inhibits fibroblast proliferation, thus targeting different stages of the PVR process. A subsequent trial in 2007, conducted by Wickham et al., explored the use of LMWH+5FU in an unselected sample of 641 patients with rhegmatogenous retinal detachments and found no significant differences in postoperative PVR rates (4.9% vs 7.0%, in treatment and placebo groups, respectively, p=0.309) but patients in the treatment group who had non-macular involving detachment had significantly worse visual acuity, presumably related to 5-FU toxicity (23). Taking into account these two studies, the choice of a pre-PVR study population may be an important factor in achieving a greater effect size and minimising the risk of visual loss with this treatment.

**Table 4:** Summary of studies that used steroids as adjunctive therapy for proliferative vitreoretinopathy.

Author, year	Sample size	Study design	Inclusion criteria	Route of administration	Steroid	Results
Acar et al., 2010 (8)	72	Case-control	PVR grade C1 or worse	Intravitreal	TA 4mg	Redetachment rates were 12.50% in the treatment group and 21.87% in controls (P = 0.349).
Ahmadieh et al., 2008 (9)	75	RCT	PVR grade C or worse	Intravitreal	TA 4mg	Retinal reattachment without any reoperation in 32 eyes was 84.2% and 78.4% in the adjunctive treatment and control groups, respectively, at 6 months (P = 0.5).
Balie E et al., 2010 (10)	34	RCT	RRD	Pre op subconjunctival	Dexamethasone diphosphate 10mg	Statistically significant decrease in laser flare measurements at the 1-week postoperative visit.
Banerjee et al., 2017 (5)	140	RCT	PVR grade C	Intravitreal	Ozurdex (slow-release dexamethasone implant) at vitrectomy and at silicone oil removal	Anatomical success was similar between the two groups (49.3% vs. 46.3%, adjunct vs. control; odds ratio, 0.89; 95% confidence interval, 0.46-1.74; P = 0.733).
Cheema et al., 2007 (6)	24	Prospective, non-comparative	PVR grade C	Intravitreal	TA 4 mg	87.5% of patients had anatomical success at the final follow-up.
Chen et al., 2011 (11)	37	Retrospective case series	PVR grade C	Intravitreal	TA 2mg at vitrectomy and 2mg at silicone oil removal	Retina was reattached in 36 (97.3%) eyes at the last visit.
Dehghan et al., 2010 (12)	52	RCT	RRD	Oral	Prednisolone 1mg/kg for 10 days	Postoperative PVR in the treatment and placebo groups 1 versus 3 (p=0.33)

RCT, randomised controlled trial; PVR, proliferative vitreoretinopathy; RRD, rhegmatogenous retinal detachment; TA, triamcinolone acetonide.

There is also evidence from animal studies for the effectiveness of steroids, given in the inflammatory phase of PVR. In a macrophage-induced rabbit model of PVR, a combination treatment of TA given immediately after PVR induction, followed by daunomycin given on day 6, was most effective in preventing retinal re-detachment from PVR (8.3%) compared to TA alone (33.3%), daunomycin alone (16.1%), and controls (83.3%) (24). In another study, using the same PVR model, TA, liposomal daunomycin, free daunomycin, and empty liposomes were injected at the point of PVR induction. PVR with retinal detachment occurred in 13.3%, 33.3%, 50%, and 77.5% of cases, respectively (25). In addition to demonstrating the effectiveness of TA given early in the disease process, the study showed that encapsulation of daunomycin in liposomes increased its effectiveness in preventing PVR compared to free daunomycin. Liposomal prednisolone phosphate, where the steroid is encapsulated within liposomes, may have a similar advantage over free triamcinolone acetonide, by prolonging half-life and avoidance of toxic effects (cataracts and glaucoma) from unintended action at the trabecular meshwork (15).

There are some limitations to our study. The sample size was small, which may limit the generalizability of the findings to a broader population. Larger sample sizes or additional animal models may provide more robust results. The study is, however, adequately powered to detect the large effect size as demonstrated in our study. In the 28-day study, we did not observe any rise in IOP or cataract formation in the eyes of all treatment groups. The short study duration limits the assessment of long-term treatment effects and potential late-onset side effects. A longer-duration study would be needed to establish whether these findings remain true since steroid-induced cataracts and ocular hypertension are well-established side effects. As this is a pilot study with the aim of demonstrating proof of efficacy, we did not design the study to evaluate the pharmacokinetics, nor assess retinal toxicity. These questions will be addressed in a subsequent study. Our study was not designed to assess the timing of treatment on efficacy, but our group had previously shown in an experimental model of PVR that the inflammatory phase peaks within the first 2 weeks of PVR induction, well before definite signs of PVR can be observed on fundoscopic examination. It can thus be inferred that anti-inflammatory therapy should be initiated as early as possible to optimize treatment outcomes. Lastly, while minipig and human eyes share many similarities, including globe size, vitreous volume, and vitreous humor properties, some differences may have an impact on the translation of findings to human disease. For example, minipig eyes do not have a macula or fovea, and chondroitin sulfate chains in the human vitreous consist of 6-sulfated disaccharides, compared to 4-sulfated disaccharides in the minipig vitreous (26).

In conclusion, we demonstrated the effectiveness of a single dose of intravitreal liposomal prednisolone phosphate in mitigating the severity of PVR in a minipig model. Liposomes as a drug delivery system for steroids could be an effective adjunctive treatment for patients with retinal detachment at high risk of developing PVR. Further studies are needed to validate these findings, optimize the dose, evaluate different routes of administration, and evaluate the potential synergistic effects of liposomal steroids with anti-proliferative therapeutics on the treatment of PVR.

## Data availability statement

The raw data supporting the conclusions of this article will be made available by the authors, without undue reservation.

## Ethics statement

The animal study was approved by Singhealth Institute Animal Care and Use Committee. The study was conducted in accordance with the local legislation and institutional requirements.

## Author contributions

CW, JB, CH, JL, SS, AV, and TW contributed to the design and conduct of the study. All authors contributed to the writing and revision of the manuscript. All authors contributed to the article and approved the submitted version.
